# Bronchial Mucosal Abnormalities in Idiopathic Subglottic Stenosis

**DOI:** 10.1002/lary.70543

**Published:** 2026-04-12

**Authors:** Sydney Ring, Andrew Jay Bowen, Tessa Griffin, J. Scott Ferguson, Seth H. Dailey

**Affiliations:** ^1^ Department of Otorhinolaryngology‐Head and Neck Surgery Mayo Clinic Rochester Minnesota USA; ^2^ Metrohealth Medical Center Cleveland Ohio USA; ^3^ Division of Otolaryngology‐Head and Neck Surgery, Department of Surgery University of Wisconsin School of Medicine and Public Health Madison Wisconsin USA; ^4^ University of Wisconsin School of Medicine and Public Health Madison Wisconsin USA; ^5^ Division of Allergy, Pulmonary, and Critical Care, Department of Medicine University of Wisconsin School of Medicine and Public Health Wisconsin USA

**Keywords:** airway inflammation, bronchial diverticula, bronchoscopy, subglottic stenosis

## Abstract

**Objectives:**

Investigate synchronous lower airway findings during bronchoscopy in idiopathic subglottic stenosis (iSGS).

**Methods:**

Retrospective analysis of bronchoscopic images from adult iSGS patients (January 2018–June 2021). Images were reviewed for pits, depressions, nodules, and striations distal to the cricotracheal junction.

**Results:**

Forty‐eight female patients; mean age 56 years. Comorbidities: gastroesophageal reflux 34%, hypertension 34%, diabetes mellitus 25%. All patients had pits and depressions; striations in 48/48, with both longitudinal and transverse in 32/48 (66.7%), transverse only in 13/48 (27.1%), and longitudinal only in 3/48 (6.3%). Spirometry available in 32; 60% showed normal or mild–moderate fixed obstruction. Four patients had FEV_1_/FVC < 0.7; two had asthma, one COPD, and one no obstructive diagnosis; none were smokers.

**Conclusion:**

Bronchial mucosal abnormalities are prevalent in iSGS and may indicate distal airway involvement. Routine flexible bronchoscopy should be considered during evaluation and surveillance.

**Level of Evidence:**

4.

## Graphical abstract

Idiopathic subglottic stenosis (iSGS) is a challenging variant of airway stenosis associated with consistent distal airway abnormalities on bronchoscopy, including pits, depressions, and striations. Despite these findings, most patients demonstrate normal or mild–moderate obstruction on spirometry. These results suggest that lower airway involvement may be underrecognized in iSGS and support the incorporation of routine bronchoscopy into evaluation and surveillance of the condition.

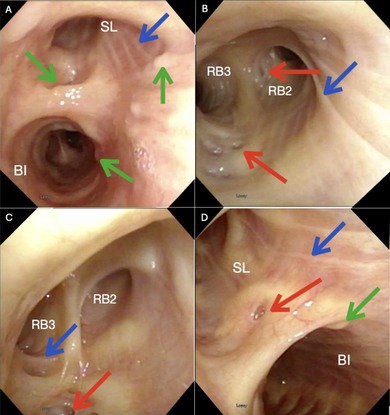

## 1 Introduction

Idiopathic subglottic stenosis (iSGS) is a rare inflammatory disease exclusively affecting the cricotracheal junction that occurs nearly exclusively in Caucasian women of Northern European descent [1]. Characterized by friable, inflammatory fibrous tissue in the subglottic region, it causes progressive airway narrowing with symptoms of dyspnea, cough, and voice change. Treatment for this disease process is surgical. The most commonly performed procedures are endoscopic dilation of the narrowed portion of the airway and excision of the friable tissue [2]. The cause of iSGS remains unknown—currently there are multiple theories proposed including: an unknown autoimmune process, bacterial infection, and even behavioral origin [3, 4, 5].

With the first reported case by Brandenburg in 1972, an abundance of literature exists discussing the anatomy, evaluation, management, and long‐term outcomes of iSGS [1, 6, 7, 8]. The classic description of iSGS is progression of fibrous tissue that is intermittently inflamed in appearance and exclusively starts at the cricotracheal junction, which, when biopsied, is negative for any identifiable pathology [9]. Glottic involvement of the disease is seldom observed in the early disease course and, if present, is suggestive of an autoimmune process such as granulomatosis with polyangiitis [10, 11]. This investigation reports on 48 adult iSGS patients who demonstrated synchronous lower airway findings that have not previously been reported with this condition. The synchronous distal mucosal findings seen during bronchoscopy may support the ‘Unified Airway Theory’ which suggests that the respiratory tract, including the nose, sinuses, throat, and bronchi, function as a single, integrated system [12]. According to this theory, inflammatory processes or diseases affecting one part of the airway can influence other parts. As such, we report findings which were seen during bronchoscopy along bronchial mucosal surfaces which we describe as pits, depressions, mucosal nodules and mucosal striations. A review of patient comorbidities and pulmonary function data is also reported.

## 2 Methods

### 2.1 Patient Population

This study was approved by the Institutional Review Board of the University of Wisconsin‐Madison (IRB# 2013‐0751) to examine patients who were treated by the senior author for iSGS. Inclusion criteria were patients older than 18 years of age with biopsy confirmed iSGS who had undergone an office trans‐nasal bronchoscopy (described below) from January 2018 to June 2021. These patients were identified from a clinical database of iSGS patients in EPIC (Epic Systems Corporation, Verona WI) that is maintained by the senior author. Patients who did not receive a bronchoscopic exam extending past the main carina were excluded from the study.

### 2.2 Transnasal Tracheoscopy With Bronchoscopy

As described previously [13], trans‐nasal tracheoscopy at this tertiary care institution is performed for iSGS patients as part of disease surveillance in addition to serving as part of a therapeutic procedure with the injection of steroid into the fibrotic tissue [14]. After local anesthesia of the nasopharynx and glottis, a lubricated channeled flexible distal chip laryngoscope (VNL‐1570STK; PENTAX) was passed through the nasal cavity along the floor of the nose into the oropharynx. After additional topical anesthesia, the endoscope was passed through the vocal folds down into the subglottis noting any scar tissue. It was then advanced down to the distal trachea and into each mainstem bronchus. After examination of the mainstem bronchi, the laryngoscope was then slowly withdrawn from the airway. Video and images captured during the examinations were uploaded immediately into the patient's electronic medical record. Therapeutic injection of steroids was performed if indicated. All procedures were performed by the senior author.

### 2.3 Bronchoscopic Images

**FIGURE 1:**
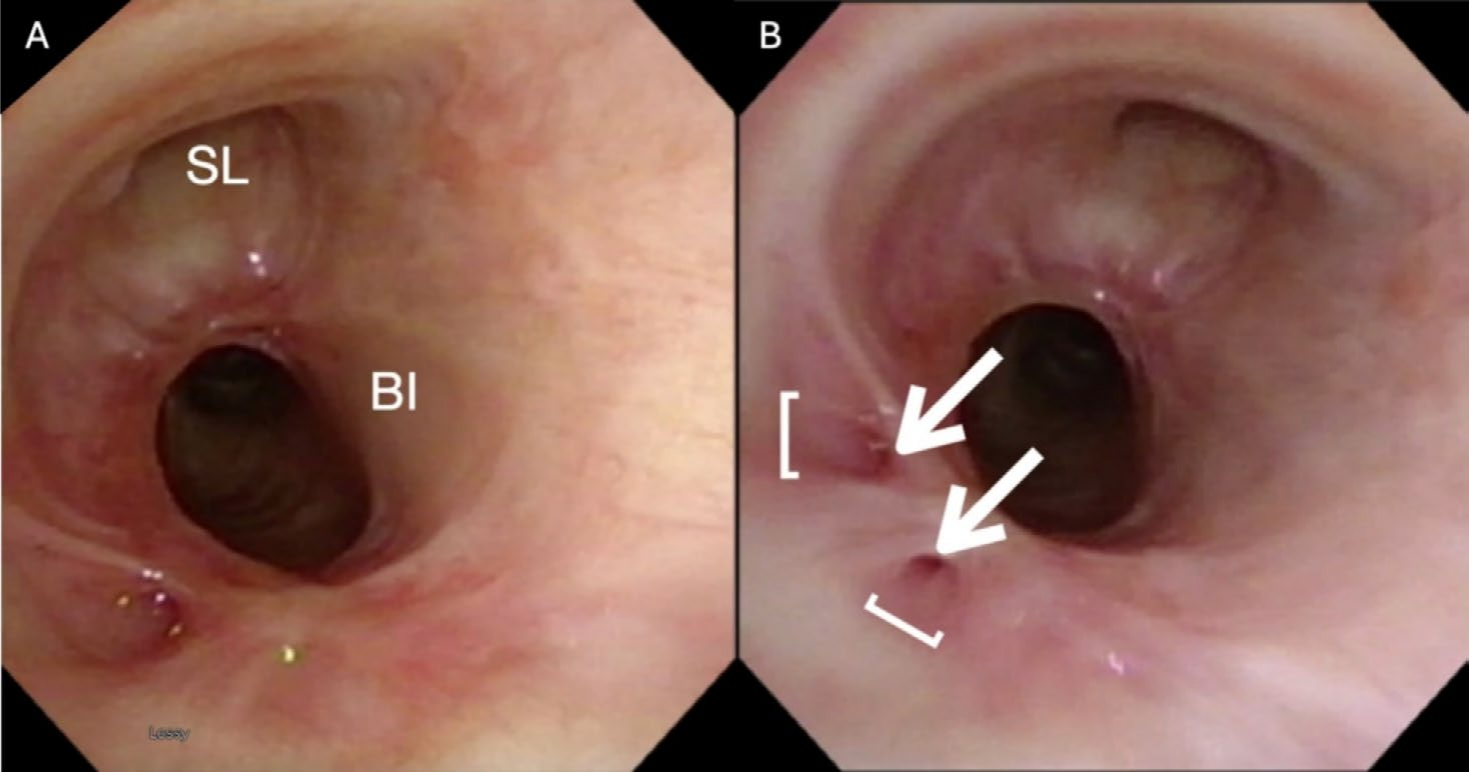
(A, B) Right superior lobar bronchus (SL) and bronchus intermedius (BI). A mucosal depression (brackets) and a mucosal pit (arrow) are visible.

**FIGURE 2:**
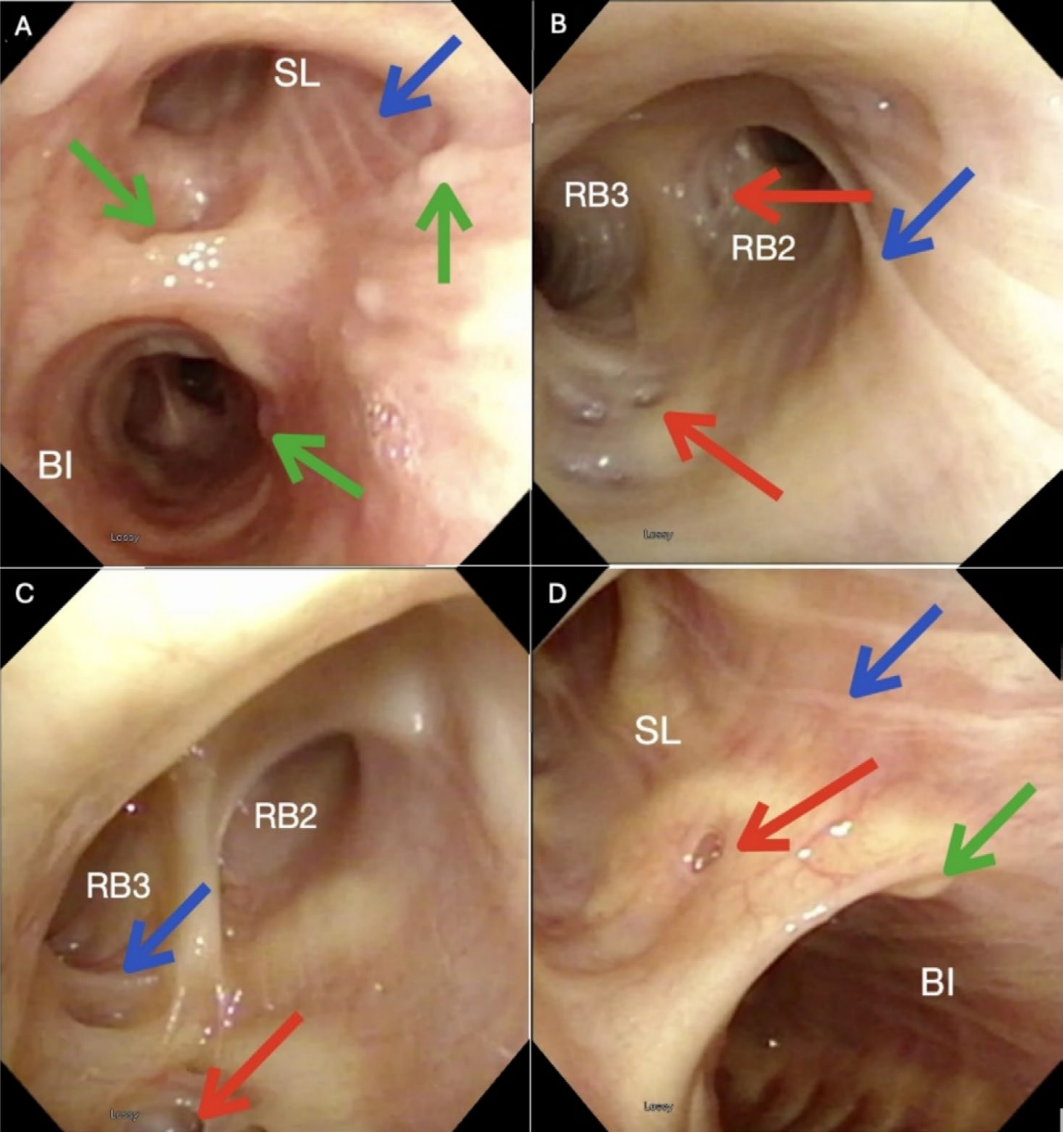
(A, B) Bronchoscopic views of the right superior lobar bronchus (SL) and bronchus intermedius (BI). Panels show bronchial pits (arrows), longitudinal striations, transverse striations, and mucosal nodules. Segmental bronchi RB2 and RB3 are labeled where applicable.

For patients that had multiple examinations extending past the carina, the earliest, rather than the best, bronchoscopy was used for consistent analysis. The endoscopists endeavored at all times to examine the trachea all the way down to and including the mainstem bronchi as per clinical pattern and protocol. Despite best efforts and a standardized topical anesthesia protocol, the extent of full visualization down to the mainstem bronchi was variable. Given the findings visualized at the level of the bronchi, we developed terms to describe our observations. We define mucosal pits as a deep pocket of mucosal tissue of varying size (Figures 1 and 2). We define mucosal depressions as shallow indentations of mucosa of varying shape and size that are adjacent to or seen separately from pits (Figure 1B). We define nodules as circumferential sessile raised areas of mucosa that are pale in contrast to the surrounding tissue under white light. We define striations as linear grooves of mucosa located outside of normal ridges otherwise observed in the bronchi (Figure 2). Striations were further defined by whether they were longitudinal (long and linear) versus transverse (short and horizontally based). All bronchoscopic images were reviewed by the same member of the study team and noted for the presence of these images.

### 2.4 Demographics and Analysis

For patients included in the analysis, basic demographic information was collected including age, sex, BMI, and medical comorbidities were recorded for each patient. Additionally, a thorough review of pulmonary function testing (PFT) with particular focus on the shape of the flow‐volume loop as described in the report and the FEV1/FVC ratio (forced expiratory volume in 1 s [FEV_1_]/forced vital capacity [FVC]) was collected. The GOLD criteria of FEV1/FVC ratio less than 0.7 for the diagnosis of COPD was utilized [15]. Given the exploratory nature of the study, descriptive statistics were used to analyze demographic information, pulmonary function tests, and bronchoscopic data.

## 3 Results

### 3.1 Retrospective Review

Sixty‐eight patients with iSGS were reviewed, of which 48 had flexible endoscopy with recorded images past the carina and were included in the analysis (Table 1). All patients were female. The mean patient age was 56 years (range 36–81 years). Mean BMI was 30.2 (range 19.6–50). Seven patients were former smokers with only one patient having quit less than 15 years before their iSGS diagnosis. The most common comorbidities in this population included gastroesophageal reflux, hypertension, and diabetes mellitus at a rate of 34%, 34%, and 25% respectively. On review of the bronchial mucosa, all patients demonstrated pits and depressions. No patient demonstrated distal bronchial stenosis or circumferential scarring in the bronchi. All patients were also observed to have striations with 32 (66.7%) having both longitudinal and transverse types. Of the patients who demonstrated either transverse or longitudinal striations solely, the most commonly isolated striation form was transverse (13 patients) compared to longitudinal (3 patients) (Table 1). Spirometry data was available for 32 patients and of these, 26 had the FEV1/FVC ratio documented (Table 1). Four patients had a FEV1/FVC ratio less than 0.7. Two of the four had asthma, one patient had a diagnosis of COPD, while the final patient with FEV1/FVC ratio of less than 0.7 did not have any documented obstructive lung disease. There was no smoking history among these 4 patients.

**TABLE 1:** Bronchoscopy findings and spirometry data and forced expiratory volume (FEV1), forced vital capacity (FVC).

PFTs (*n* = 68)	
Normal	10
Mild to moderate fixed obstruction	9
Severe obstruction	1
Depressed/truncated inspiratory loop	4
Depressed/truncated expiratory loop	2
Depressed/truncated both loops	4
FEV1/FVC (*n* = 26)	
Range, total	0.59–0.88
< 0.7	4
≥ 0.7	22
Bronchoscopy findings (*n* = 48)	
Pits or depressions	48
Striations	48
Both	32
Transverse	13
Longitudinal	3

Abbreviations: FEV1, First Second and Forced Expiratory Volume; FVC, Forced Vital Capacity.

## 4 Discussion

iSGS remains a perplexing airway disease. On the one hand, it demonstrates remarkable homogeneity, almost exclusively affecting otherwise healthy, perimenopausal women [1, 16]. On the other hand, it displays striking heterogeneity in its clinical behavior, with significant variability in both recurrence rates and overall aggressiveness. This paradox underscores the poorly understood pathophysiology of iSGS. The disease is classically defined by inflamed fibrous tissue arising along the posterolateral subglottis, localized to the cricotracheal junction between the cricoid cartilage and first tracheal ring. The consistent restriction of disease to this anatomic site, without other organ system involvement, serves as an important differentiator from autoimmune conditions such as granulomatosis with polyangiitis [10].

In the present study, we describe abnormal mucosal changes within the bronchi that were identified during flexible bronchoscopy performed concurrently with transnasal tracheoscopy in patients with iSGS. To our knowledge, these findings have not previously been reported in this population, expanding the known anatomic distribution of airway changes associated with the disease.

Although novel in the context of iSGS, similar mucosal abnormalities have been well described in chronic lung diseases, including chronic bronchitis, bronchiectasis, and COPD [17, 18, 19, 20, 21, 22, 23]. Bronchial pits were originally reported histologically by Duprez in 1953 as dilated ducts of bronchial glands that had undergone cystic degeneration, giving rise to small diverticula visible on bronchograms before the widespread adoption of flexible bronchoscopy [17]. Mucosal pits were often accompanied by transverse or longitudinal striations, with deeper pits extending into the lateral wall or base of shallow depressions [17, 22]. These established features closely paralleled those observed in our cohort (Figures 1 and 2, Figures S1–S5). Histologic and ultrastructural studies have demonstrated that longitudinal striations are composed primarily of elastic fibers, while transverse striations contain myofibrils [22]. The overlying mucosa in these regions is typically denuded epithelium with a thickened basal lamina and thin lamina propria. This weakened surface tissue sinks between the interwoven elastic and myofibrillar lattices, creating the depressions visualized endoscopically. Consistent with prior reports, Wang and Duprez also noted that regions just distal to bronchial bifurcations were more affected than inter‐bifurcation areas, with distal bronchi showing greater involvement than proximal bronchi [17, 22]. Our findings mirrored these observations, as lesions were commonly identified adjacent to the right mainstem carina or at the bifurcation of the right upper lobe segmental bronchi.

The resemblance between our cohort and patients with chronic inflammatory lung disease raises the possibility that the pathologic process in iSGS extends beyond the cricotracheal junction to involve the distal bronchial mucosa. In this framework, the bronchial pits, striations, and nodules we observed may represent structural manifestations of an ongoing inflammatory process, aligning with the ‘Unified Airway Theory.’ [12, 24, 25] According to this paradigm, the respiratory tract functions as a single immunologic and physiologic unit, whereby inflammatory responses in one region can modulate activity in another. This concept, well established in allergic rhinitis, asthma, and chronic rhinosinusitis, provides a biologically plausible model for lower airway involvement in iSGS.

At the same time, alternative explanations must be considered. The observed bronchial lesions may not be inflammatory in origin, but instead mechanical. One possible mechanism is the formation of pulsion diverticula, generated by abnormal tracheobronchial airflow dynamics in iSGS patients. Turbulent airflow through a narrowed subglottic segment, coupled with elevated intraluminal pressure or repetitive shearing forces at bronchial bifurcations, could create localized stress points along the mucosa. Over time, these stresses may produce small diverticula, depressions, or striations independent of any inflammatory pathway. If true, these lesions would reflect a biomechanical consequence of altered flow patterns rather than a direct extension of iSGS pathology.

The distribution of lesions provides further insight. Prior studies have shown that asymptomatic individuals can exhibit small proximal tracheal diverticula [26, 27, 28], while pathologic diverticuli are more often identified in the distal bronchi [29, 30, 31, 32]. This difference likely reflects underlying variations in airway wall architecture and mechanical forces. Proximal lesions are often attributed to congenital weakness or mucous gland herniation, whereas distal diverticula may develop in response to chronic stressors such as turbulence, coughing, or inflammation. In iSGS, the narrowed cricotracheal segment could amplify distal shear forces and intrabronchial pressures, predisposing to lesions at physiologically complex sites such as the carina or upper lobe bifurcations.

Furthermore, it is reasonable to expect that a process affecting the lower airway may be related to that affecting the subglottis, between mechanical and inflammatory contributions. Despite these theories, progressive bronchial stenosis and post‐obstructive complications are rarely reported in these patients. Despite the smaller size of the lower airways, rapid circumferential closure does not occur due to more retention of the resilient epithelial barrier in the lower airways [1]. The pathologic fibroblast subtypes and profibrotic Schwann cells identified in scar tissue are more concentrated in the subglottic scar tissue as well [26].

Nevertheless, the possibility that these lesions are incidental cannot be excluded. Bronchial diverticulosis has been observed in otherwise healthy individuals [27, 28, 29], and subtle depressions or striations may represent normal anatomic variants. Notably though, on thin‐cut CT images, there are reports of up to 45.5% prevalence of bronchial diverticula in non‐pathological lungs of smokers. This may represent a repetitive airway injury, or again, may be purely incidental [30]. The high prevalence in our cohort may reflect a true disease association, but it is also possible that these findings were over‐interpreted in the context of close bronchoscopic scrutiny. For this reason, we view these observations as hypothesis‐generating rather than diagnostic, warranting prospective investigation; it is entirely possible future studies may demonstrate normal variants.

Of note, although bronchial pits and striations are well established in chronic bronchitis, bronchiectasis, and COPD, the mucosal nodules observed in some iSGS patients are less clearly described outside of an association with active smoking [19, 23]. Their presence in our predominantly nonsmoking cohort raises the possibility that they represent a distinct airway structural component accentuated by mucosal atrophy. Importantly, only four patients demonstrated obstructive patterns on spirometry—one with COPD and two with asthma—and only seven of 48 patients had a smoking history. These findings suggest that the observed bronchial abnormalities in our cohort are unlikely to be explained by confounding comorbid lung disease or smoking exposure.

Given the parallels between our findings and those reported in chronic inflammatory airway conditions, the possibility that fibro‐inflammatory mechanisms are at play remains compelling. Noxious stimuli such as cigarette smoke are well‐established drivers of bronchial inflammation in COPD, whereas the trigger for iSGS remains elusive [23]. Emerging evidence implicates pathologic fibroblasts in amplifying IL‐17A signaling within the subglottis [33, 34]. It is conceivable that bronchial mucosa contributes to this inflammatory milieu, reinforcing the notion of the airway as an integrated immunologic system.

Taken together, our findings suggest that lower airway involvement may occur in iSGS, whether as a direct inflammatory extension, a secondary mechanical phenomenon, or an incidental anatomic variant.

The clinical and prognostic significance of these lesions remains uncertain. Nonetheless, their high prevalence in this series supports the routine incorporation of flexible bronchoscopy into the evaluation and surveillance of iSGS patients, both to document potential lower airway involvement and to provide a framework for future prospective studies.

This study has several limitations. First, it was retrospective and restricted to bronchoscopies performed between 2018 and 2021. Each patient contributed only a single examination, preventing assessment of lesion progression over time. Second, bronchoscopies were performed at variable points in the disease course, limiting our ability to evaluate the prognostic significance of these findings for scar recurrence. Third, we did not have a control group. Previous studies have shown histologically normal distal airways in patients with iSGS, but calcified stenoses extending to the bronchi have been frequently noted in autoimmune processes like relapsing polychondritis [35]. A case–control design would offer valuable insight into these preliminary findings.

## 5 Conclusion

To our knowledge, this is the first report of iSGS patients with bronchial findings typically seen in patients with chronic lung disease. These lesions were prevalent in the patient cohort, with bronchial pits and depressions observed in all included patients, and striations seen in the majority as well. These lesions were seen generally at the level of the carina or within the right mainstem bronchus which differs from where these lesions are identified in the general population. Our findings suggest that the inflammatory changes seen classically with iSGS may go beyond the cricotracheal junction and involve the distal airways. Given these findings, flexible bronchoscopy should be performed as part of the initial fiberoptic evaluation of the airway and on subsequent surveillance examinations. More investigation into these lesions will determine the prognostic significance of these observations.

## Funding

The authors have nothing to report.

## Ethics Statement

Approved by the University of Wisconsin–Madison IRB (2013‐0751).

## Consent

Consent waived due to retrospective design.

## Conflicts of Interest

The authors declare no conflicts of interest.

## Supporting information


**Figure S1:** (A) Normal subglottis; (B) View of a normal Right Superior Lobar Bronchus (SL) and the Bronchus Intermedius.
**Figure S2:** (A) Subglottis with residual Kenalog from injection (asterisk). (B) View of the Right Lobar Bronchus Intermedius (BI). Bronchial Pits (Red Arrow), Transverse striations (Blue Arrow).
**Figure S3:** (A) Subglottis of patient with residual Kenalog from prior injection (asterisk); (B) View of the Right Superior Lobar Bronchus (SL) and the Bronchus Intermedius (BI) with Transverse Striations (Blue Arrow).
**Figure S4:** (A) Subglottis; (B) Through the Right Superior Lobar Bronchus (SL) with views and the Anterior (RB3) and Posterior (RB2) segmental bronchi as well as the Bronchus Intermedius. Bronchial Pits (Red Arrow), Longitudinal striations (Blue Arrow).
**Figure S5:** (A) Subglottis; (B) Through the Right Superior Lobar Bronchus with views of Anterior (RB3) and Posterior (RB2) segmental bronchi as well as the Bronchus Intermedius. Bronchial Pits (Red Arrow), Transverse striations (Blue Arrow).

## Data Availability

The data that support the findings of this study are available from the corresponding author upon reasonable request.
