# Supplementary material for: Bronchial Mucosal Abnormalities in Idiopathic Subglottic Stenosis
Source: Laryngoscope. 2026 Apr 12;136(8):3527–33. doi: 10.1002/lary.70543 (PMC13357366; doi:10.1002/lary.70543)
Supplement: Supplementary file 1 — Figure S1: (A) Normal subglottis; (B) View of a normal Right Superior Lobar Bronchus (SL) and the Bronchus Intermedius. Figure S2: (A) Subglottis with residual Kenalog from injection (asterisk). (B) View of the Right Lobar Bronchus Intermedius (BI). Bronchial Pits (Red Arrow), Transverse striations (Blue Arrow). Figure S3: (A) Subglottis of patient with residual Kenalog from prior injection (asterisk); (B) View of the Right Superior Lobar Bronchus (SL) and the Bronchus Intermedius (BI) with Transverse Striations (Blue Arrow). Figure S4: (A) Subglottis; (B) Through the Right Superior Lobar Bronchus (SL) with views and the Anterior (RB3) and Posterior (RB2) segmental bronchi as well as the Bronchus Intermedius. Bronchial Pits (Red Arrow), Longitudinal striations (Blue Arrow). Figure S5: (A) Subglottis; (B) Through the Right Superior Lobar Bronchus with views of Anterior (RB3) and Posterior (RB2) segmental bronchi as well as the Bronchus Intermedius. Bronchial Pits (Red Arrow), Transverse striations (Blue Arrow). [file LARY-136-3527-s001.docx]

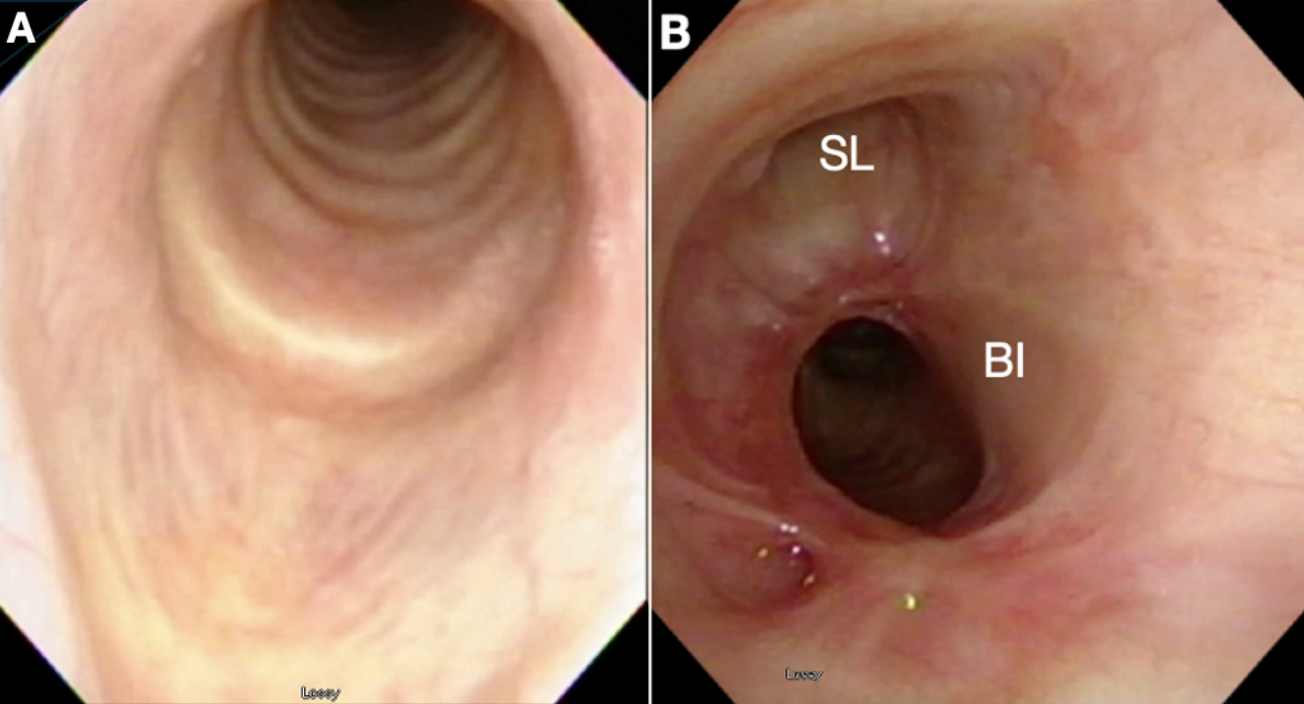


**Supplemental Figure 1: A)** Normal subglottis; **B)** View of a normal Right Superior Lobar Bronchus (SL) and the Bronchus Intermedius


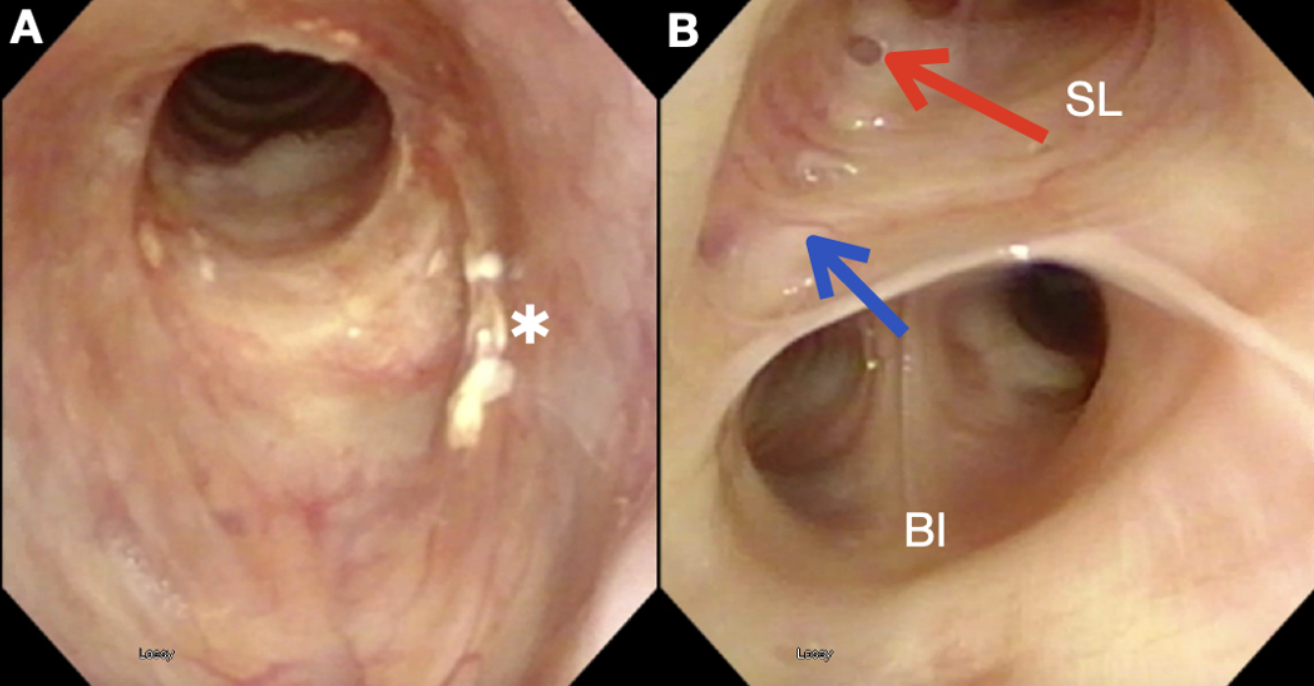


**Supplemental Figure 2: A)** Subglottis with residual Kenalog from injection (asterisk). **B)** View of the Right Lobar Bronchus Intermedius (BI). Bronchial Pits (Red Arrow), Transverse striations (Blue Arrow).


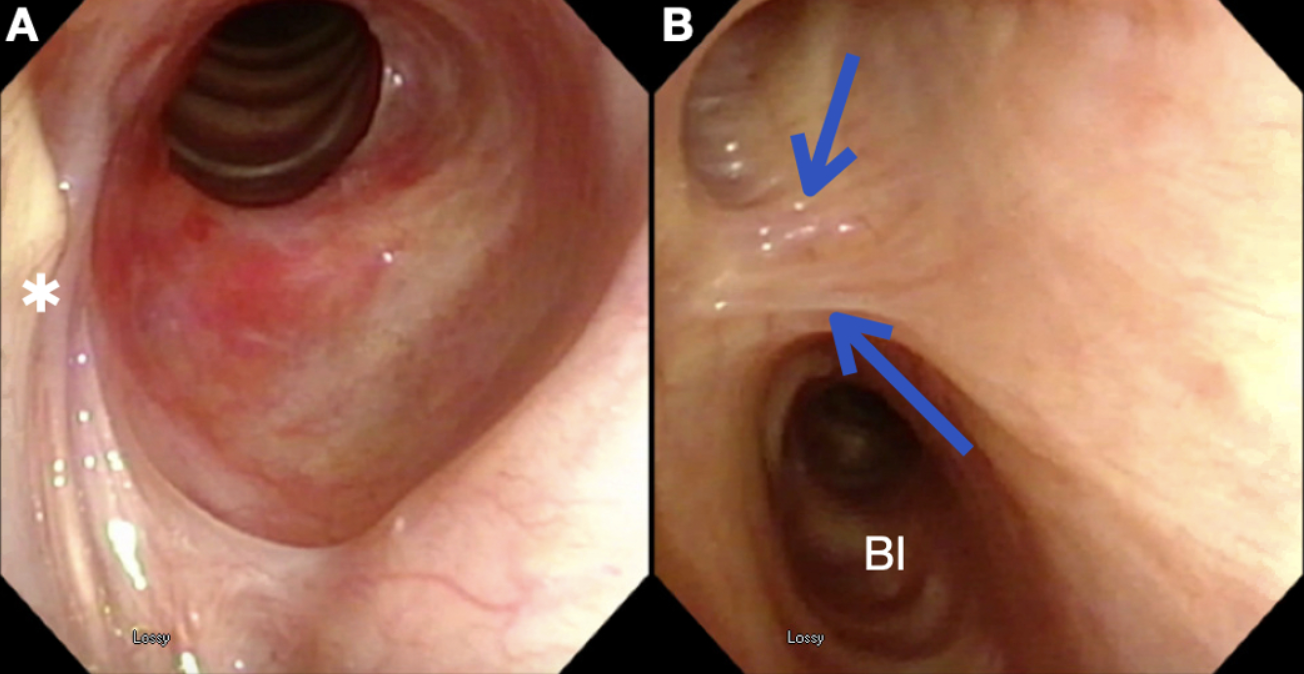


**Supplemental Figure 3: A)** Subglottis of patient with residual Kenalog from prior injection (asterisk); **B)** View of the Right Superior Lobar Bronchus (SL) and the Bronchus Intermedius (BI) with Transverse Striations (Blue Arrow).


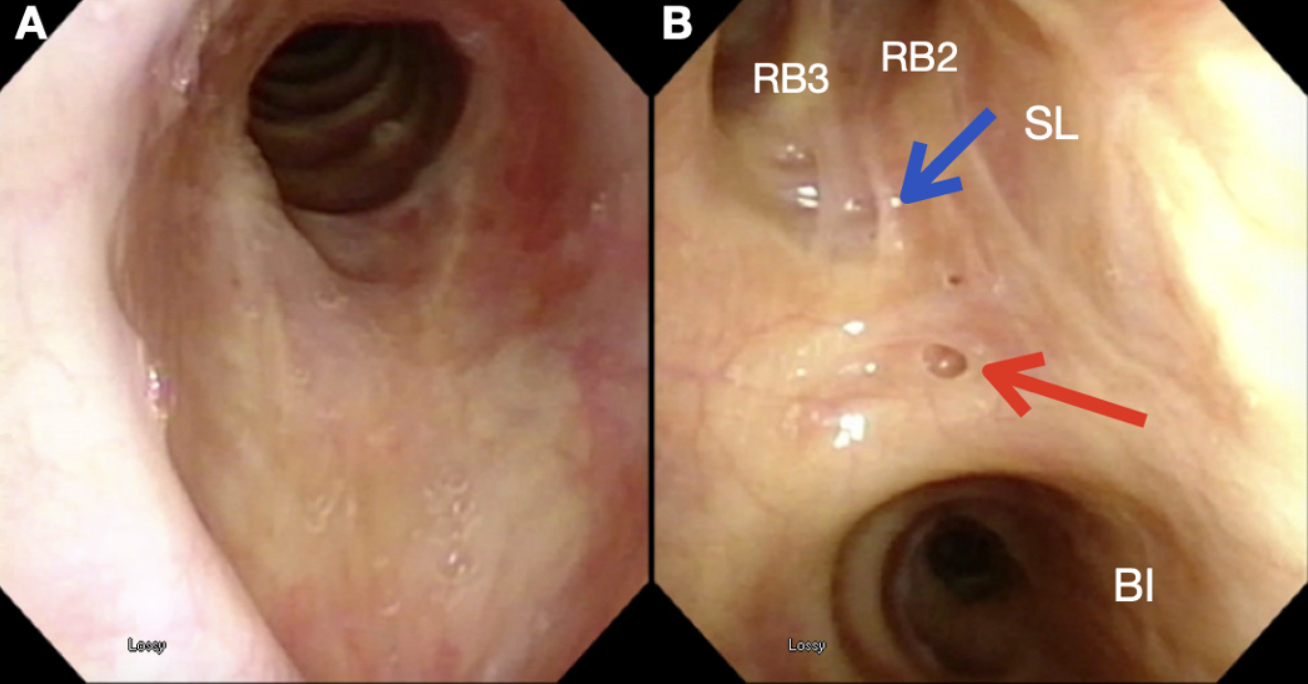


**Supplemental Figure 4: A)** Subglottis; **B)** Through the Right Superior Lobar Bronchus (SL) with views and the Anterior (RB3) and Posterior (RB2) segmental bronchi as well as the Bronchus Intermedius. Bronchial Pits (Red Arrow), Longitudinal striations (Blue Arrow).


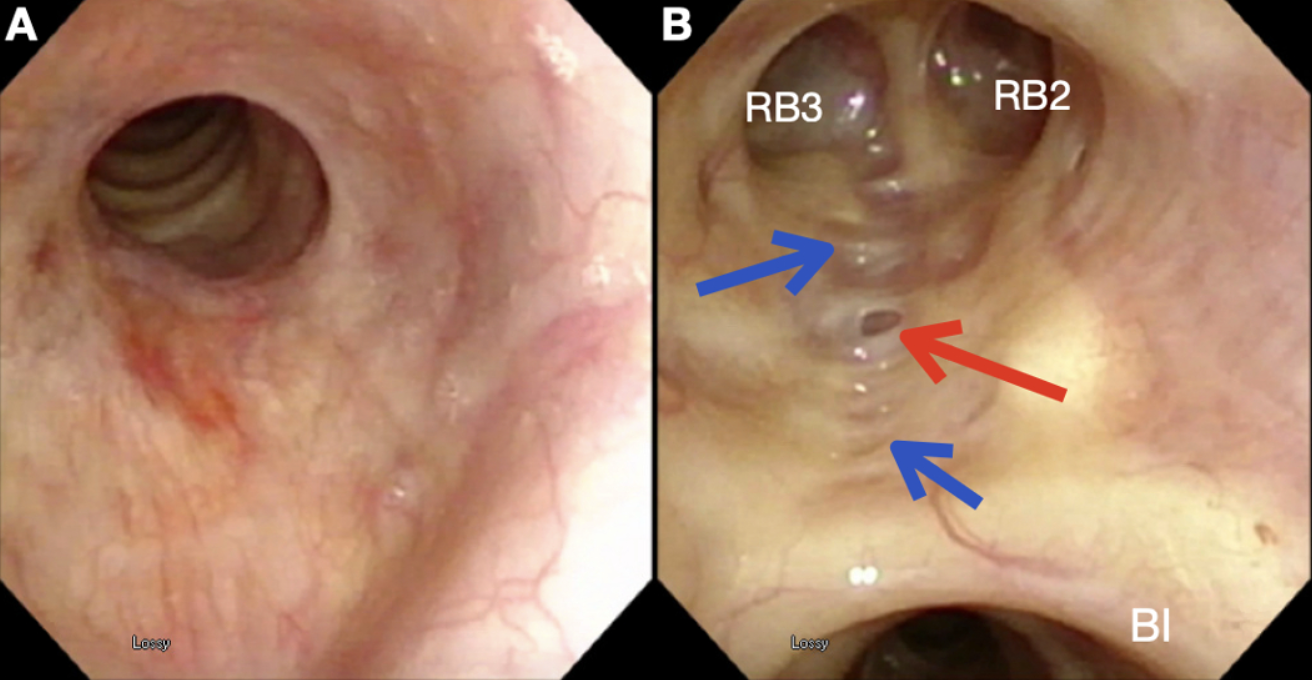


**Supplemental Figure 5: A)** Subglottis; **B)** Through the Right Superior Lobar Bronchus with views of Anterior (RB3) and Posterior (RB2) segmental bronchi as well as the Bronchus Intermedius. Bronchial Pits (Red Arrow), Transverse striations (Blue Arrow).
